# Editorial: Enzyme-Based Smart Materials

**DOI:** 10.3389/fchem.2021.815071

**Published:** 2021-12-13

**Authors:** Lei Wang, Yijing Liu, Paul E. D. Soto Rodriguez

**Affiliations:** ^1^ School of Chemistry and Chemical Engineering, Harbin Institute of Technology, Harbin, China; ^2^ Hubei Key Laboratory of Bioinorganic Chemistry and Materia Medica, Hubei Engineering Research Center for Biomaterials and Medical Protective Materials, School of Chemistry and Chemical Engineering, Huazhong University of Science and Technology (HUST), Wuhan, China; ^3^ Aix-Marseille University and Institute of Biosciences and Biotechnologies, CEA Cadarache, Marseille, France

**Keywords:** enzyme, smart materials, nanozyme, enzyme-responsive materials, medical applications

**FIGURE 1:**
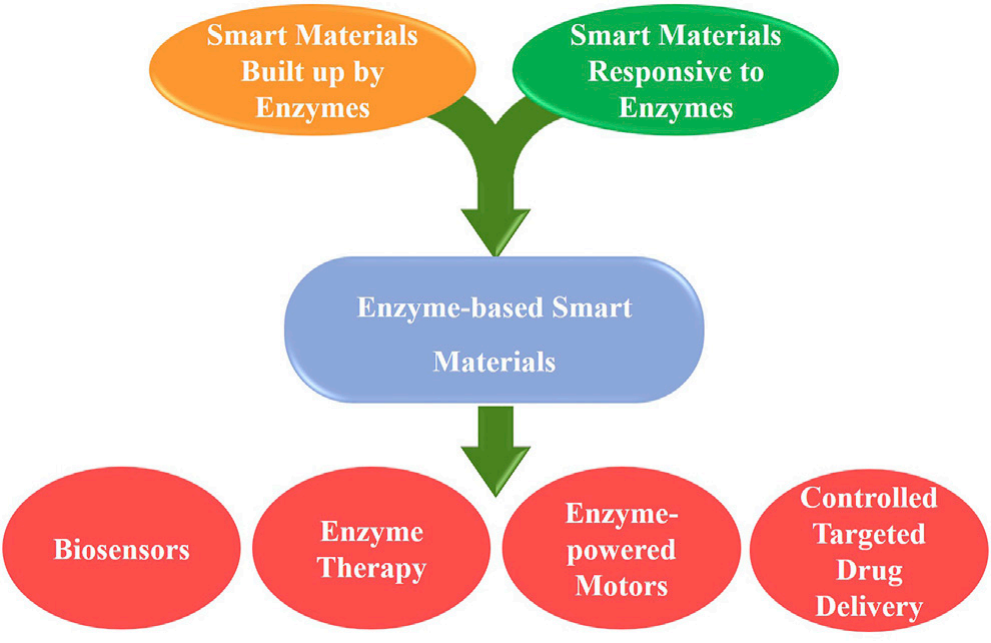
Focuses of this editorial on enzyme-based smart materials.

Smart materials, also called intelligent or stimuli-responsive materials, are essential for developing next-generation micro-/nanodevices toward biomedical applications. Smart materials chosen for bioapplications need to be biocompatible. Therefore, enzymes with excellent catalytic properties become good candidates for designing smart materials. Generally, enzyme-based smart materials allow for two-way communication between the biological environment and the material, which resembles the dynamics of natural biological materials, and also provide a nature-inspired strategy for designing enzyme-responsive materials: 1) smart materials built up by enzymes, and 2) smart materials responsive to enzymes (Figure 1). Therefore, this special research topic focuses on the enzyme-based materials applied in the biomedical areas.

In principle, the enzymes need to maintain high catalytic properties under working conditions. However, the intrinsic fragile nature of enzymes makes them prone to denaturation or destabilization when working under harsh circumstances, leading to unavoidably shortened lifespan and extremely high cost. One of the focuses is bio-enzymes encoded by antibiotic resistance genes (ARGs). Their enzyme activity might be influenced by the co-selective pressure of ARGs and heavy metal pollution in the soil in the context of heavy metal contents and the relative abundance of ARGs. Qi et al. investigated the distribution characteristics and the co-selective relationship of 28 ARGs and eight heavy metals in soil in a dairy farm *via* the geographic information system technique, providing a visual insight into correlations between distribution of typical heavy metals and ARGs, toward the understanding of representative heavy metals on the properties of ARGs, as well as the activity of certain bio-enzymes.

To enhance the catalytic performance and stability, one of the traditional and prevalently recognized methods is enzyme immobilization. Based on this idea, Yuan et al. prepared lysozyme-immobilized ZnO nanoparticles, exhibiting the synergistic antibacterial effects against *Escherichia coli* and *Staphylococcus aureus* by the mechanism of reactive oxygen species (ROS) generation, which had better performance than either pure ZnO nanoparticles or pure lysozyme both *in vitro* and *in vivo*.

Besides nanoparticles, hydrogels could also be a suitable substrate for enzyme loading. In doing so, Yao et al. engineered a ClyC-loaded alginate hydrogel (ClyC-AH) to improve the continuous therapeutic outcome against *Staphylococcus aureus*, based on the sustained release of ClyC with good stability and activity. Notably, compared to pure ClyC, the use of ClyC-AH improved its biocompatibility and adequate time, thus providing a promising future in the *Staphylococcus aureus*–targeting therapy.

In addition to enzyme immobilization, developing other nanomaterials with enzyme-like properties could be another solution. A new type of nanomaterials termed nanozymes has attracted significant research attention, owing to their high stability, easy surface modification, adjustable activity by size/structure/components, facile preparation, and preservation, thus becoming the potentially ideal substitute for natural enzymes in various bioapplications. Nanozymes could be cataloged into three kinds: metal-oxide nanozymes, noble metal nanozymes and carbon-based nanozymes. There are various applications of nanozymes in the biomedical field, including biosensing, enzyme-based therapy, antibacterial property, and anti-inflammatory property, as summarized by Wang et al. In this field, metal-related nanozymes own advantages in bioanalysis, such as Pd-Ir materials. However, regarding the preparation of nanozymes based on Pd-Ir nanocubes, it is a technical challenge to deposit atomic layers of Ir on the surface of Pd nanocubes due to the relatively low energy barrier of homogeneous nucleation of Ir atoms, compared to heterogeneous nucleation. To solve this problem, Li et al. synthesized Pd-Ir nanocubes with an Ir shell of atomic thickness in an aqueous solution at room temperature. In this way, biomolecules such as antibodies and nucleic acids have free access to the surface of Pd-Ir nanozymes, facilitating these cubic nanozymes for biosensing based on their excellent peroxidase activity and fluorescence quenching ability, showing great potential in clinical applications.

Moreover, being one primary type of nanozymes, carbon-based nanozymes possess unique virtue compared to metal-related nanozymes because of their excellent water dispersion, stable chemical inertness, high photobleaching resistance, and superior surface engineering. Thereby, many investigations have been conducted in biomedicine, catalysis, and biosensing fields based on carbon dot–based nanozymes, which was reviewed by Jin et al., offering deep insights into solving the dilemma of easy inactivation of natural enzymes.

Two components should be included regarding smart materials responsive to enzymes: 1) an enzyme-sensitive component, like substrates or substrate mimics; 2) one component controlling the material changes. Thereby, enzyme-responsive biomaterials possess several advantages over other materials because of their selective catalytic reactions, mild or biological working conditions, and vital functions in healthy and diseased biological pathways. Therefore, enzyme-responsive materials have been successfully applied in various biomedical fields. For instance, photodynamic therapy is a mini-invasive tumor therapy based on ROS induced by photosensitizers. However, the non-specific distribution of photosensitizers would potentially be harmful to the healthy tissues, thereby inhibiting their practical applications. To address this issue, Liu et al. summarized the enzyme-based smart materials responsive to the unique enzymatic tumor environment for the targeted drug delivery to improve therapeutic effects, to avoid side effects, and to further boost the development of PDT in the treatment of malignancies.

